# Age-related differences in treatment strategies and clinical outcomes in unselected cohort of patients with ST-segment elevation myocardial infarction transferred for primary angioplasty

**DOI:** 10.1007/s11239-012-0713-y

**Published:** 2012-03-29

**Authors:** Artur Dziewierz, Zbigniew Siudak, Tomasz Rakowski, Jacek S. Dubiel, Dariusz Dudek

**Affiliations:** 1 2nd Department of Cardiology, Jagiellonian University Medical College, Krakow, Poland; 2 Department of Interventional Cardiology, Jagiellonian University Medical College, Kopernika 17 Street, 31-501 Krakow, Poland

**Keywords:** Myocardial infarction, Angioplasty, Percutaneous coronary intervention, Elderly, Registries, Outcomes

## Abstract

Data concerning the benefits and risks of primary PCI in the elderly patients presenting with ST-segment elevation myocardial infarction (STEMI) are limited. Thus, the objective of the study was to assess age-dependent differences in the treatment and outcomes of STEMI patients transferred for primary PCI. Data were gathered on 1,650 consecutive STEMI patients from hospital networks in seven countries of Europe from November 2005 to January 2007 (the EUROTRANSFER Registry population). Patients <65, 65 to 74, 75 to 84, and ≥85 years of age comprised 49.3, 27.5, 20.2, and 3 % of the registry population, respectively. Elderly patients were higher risk individuals and have experienced longer delays to reperfusion than their younger counterparts and were more likely to be treated conservatively after coronary angiography. Despite similar frequency of TIMI 3 flow before PCI, elderly patients were less likely to achieve TIMI 3 flow and ST-segment resolution >50 % after PCI, and were more likely to have PCI complications. The rates of death at 30 days, as well as at 1 year were increased with age. In the Cox regression analysis model age was an independent predictor of 30-day mortality. A trend toward higher risk of major bleeding requiring transfusion was observed. Age was an important determinant of treatment strategies selection and clinical outcomes in the group of consecutive STEMI patients transferred for primary PCI. Further efforts should be made to reduce delays and to optimize treatment of STEMI, regardless of patients’ age.

## Background

Elderly patients presenting with ST-segment elevation myocardial infarction (STEMI) are less likely to receive reperfusion therapies, both fibrynolysis and primary percutaneous coronary intervention (PCI) [1–3]. Common reasons for excluding older patients from reperfusion therapy are their delayed presentation and atypical symptoms. Also, up to 9 % of elderly patients have absolute contraindication to fibrynolytic therapy [4]. Nowadays, primary PCI is the preferred method of reperfusion for STEMI, also in elderly patients [5]. It has been shown to be more effective than fibrynolysis in reduction of ischemic events in patients ≥75 years old with STEMI with chest pain <6 h [6]. However, primary PCI carries a decreased success rate and an increased procedural risk in older patients when compared with younger ones. Elderly STEMI patients are also at the higher risk of death or other adverse ischemic and non-ischemic events as result of higher prevalence of comorbidities.

Elderly patients with STEMI are often excluded from randomized clinical trials, thus it is hard to generalize expected outcomes from randomized clinical trials to the real life setting. More reliable data on treatment and outcomes of elderly patients with STEMI can be extracted from multicenter registries. The objective of the present study was to assess whether there exist age-dependent differences in the clinical characteristics, treatment strategies and clinical outcomes in patients with STEMI transferred for primary PCI based on data from the European Registry on Patients with ST-Elevation MI Transferred for Mechanical Reperfusion with a Special Focus on Upstream Use of Abciximab (EUROTRANSFER) Registry [7–9].

## Methods

The details of the EUROTRANSFER Registry (ClinicalTrials.gov number NCT00378391) protocol and main results have been previously published [7–9]. In this registry data concerning treatment and outcomes of 1,650 consecutive, transferred STEMI patients in 15 STEMI hospital networks from 7 European countries between November 2005 and January 2007 were collected. For the purpose of this analysis patients were divided into four age groups (<65, 65–74, 75–84 and ≥85 years of age). The study protocol and execution complied with the Declaration of Helsinki and has been approved by the Institutional Review Board.

All-cause death, reinfarction and urgent revascularization (PCI or coronary artery bypass grafting) and bleeding complications: puncture site hematoma, intracranial hemorrhage, major bleeding requiring transfusion were evaluated during 30-day follow-up [7]. Additionally 1-year mortality was assessed [8]. Data concerning Thrombolysis In Myocardial Infarction (TIMI) flow in the infarct-related artery before and after PCI, ST-segment resolution after PCI, and rate of PCI complications (no-reflow, distal embolization, side branch occlusion, artery perforation) were also provided.

Data were analyzed according to the established standards of descriptive statistics. Results were presented as percentages of patients or medians (inter-quartile range). Differences in dichotomous variables were analyzed using Chi-square test and the Fisher’s exact test as appropriate. Continuous variables were compared by the Kruskal–Wallis test. The difference in death rates between groups during follow-up period was assessed by the Kaplan–Meier method using the log-rank test. In addition, multivariate Cox regression analysis was performed to find significant predictors of 30-day death. Risk of 30-day death was expressed as hazard ratios with 95% confidence intervals. All tests were two-tailed and a *p* value of <0.05 was considered statistically significant. All statistical analysis was performed using SPSS 15.0 (SPSS Inc., Chicago, IL).

## Results

Data on 1,650 patients were entered into the EUROTRANSFER Registry database. Patients <65, 65–74, 75–84, and ≥85 years of age comprised 49.3, 27.5, 20.2, and 3.0 % of the registry population, respectively. Characteristics of patient population according to age are shown in Table 1. The prevalence of female gender, diabetes mellitus, previous myocardial infarction, previous heart failure symptoms, previous stroke, current smoking and chronic kidney disease, as well as body mass index and diastolic blood pressure changed across age groups.

**Table 1:** Baseline demographics and clinical status on admission to percutaneous coronary intervention center stratified by age

Variable	Age (years)				*p* value
	<65 (*n* = 814)	65–75 (*n* = 454)	75–85 (*n* = 333)	≥85 (*n* = 49)	
Male	82.1	70.3	54.7	40.8	<0.0001
Body mass index (kg/m^2^)	26.9 (24.4–29.9)	27.1 (24.8–29.7)	25.7 (23.5–28.4)	24.1 (22.4–26.7)	<0.0001
Diabetes mellitus	11.9	18.7	21.9	14.3	<0.0001
Previous myocardial infarction	8.6	16.1	16.8	18.4	<0.0001
Previous heart failure symptoms	0.4	1.8	3.3	0	0.001
Previous percutaneous coronary intervention	6.9	7	9	6.1	0.65
Previous coronary artery bypass grafting	0.9	2.4	0.9	2	0.094
Previous stroke	2.2	4.8	4.2	8.2	0.013
Current smoker	55	22.9	12.9	2	<0.0001
Peripheral arterial disease	2.7	3.5	3	10.2	0.064
Chronic kidney disease	1.0	2.9	3.9	4.1	0.004
Heart rate on admission (beat/min)	78 (68–88)	78 (68–90)	76 (65–88)	75 (65–90)	0.50
Systolic blood pressure on admission (mmHg)	130 (150–120)	126 (117–156)	135 (115–152)	134 (116–166)	0.58
Diastolic blood pressure on admission (mmHg)	80 (70–91)	80 (70–90)	75 (65–85)	80 (64–90)	<0.0001
Killip IV on admission	2.7	3.3	3.3	6.1	0.78

Values are presented as percentages or medians (inter-quartile range)

Data concerning pharmacological and interventional treatment are summarized in Table 2. Elderly patients has experienced longer delays to individual stages of treatment than their younger counterparts (Table 2) and they were less likely to be treated with diagnosis-to-balloon time <90 min (for age <65, 65–74, 75–84, and ≥85 percentage of patients with diagnosis-to-balloon time <90 min was as follows: 40.8, 34.1, 35.1, 34.7 %, *p* = 0.079). There was no difference in the frequency of unfractionated heparin and abciximab use across age groups, both in pre-cathlab and in the cathlab setting. However, a trend toward less frequent administration of clopidogrel in older patients was observed (Table 2).

**Table 2:** Concomitant medications, timing information and invasive treatment details stratified by age

Variable	Age (years)				*p* Value
	<65 (*n* = 814)	65–75 (*n* = 454)	75–85 (*n* = 333)	≥85 (*n* = 49)	
Pain-to-diagnosis time (min)	96 (52–198)	111 (60–213)	132 (68–240)	97 (47–303)	<0.0001
Diagnosis-to-balloon time (min)	100 (77–138)	109 (83–155)	109 (80–145)	110 (67–154)	0.010
Admission to cathlab-to-balloon time (min)	30 (20–41)	31 (21–45)	32 (23–44)	33 (20–47)	0.11
Pain-to-balloon time (min)	216 (150–329)	240 (165–377)	251 (168–379)	200 (155–425)	<0.0001
Clopidogrel pre-cathlab	33.7	32.8	28.8	22.4	0.19
Clopidogrel in the cathlab	46.7	46.9	48.3	36.7	0.38
Unfractionated heparin pre-cathlab	68.2	67.2	69.4	69.4	0.93
Unfractionated heparin in the cathlab	61.3	63.7	61.9	51	0.37
Abciximab pre-cathlab	43.5	46.9	42.9	34.7	0.12
Abciximab in the cathlab	23.5	21.2	17.7	26.5	
No abciximab	33	31.9	39.4	38.8	
Femoral access	86.4	84.4	88.3	87.8	0.45
IRA in baseline angiography					
LMCA	1.2	0.2	0.6	2	0.15
LAD	42.1	45.2	48	46.9	0.30
LCX	13.3	11.9	11.1	10.2	0.71
RCA	42.4	40.1	38.1	40.8	0.59
Multi-vessel disease	42.5	55.1	64.3	75.5	<0.0001
Abandoned PCI	5.7	5.5	10.2	16.3	0.002
Immediate PCI	94.3	94.5	89.8	83.7	
Number of patients undergoing immediate PCI (n)	768	429	299	41	
Stent	94.8	93.5	88.6	82.9	0.001
Drug-eluting stent	27.9	24.5	19.7	9.8	0.005
>1 stent in IRA	21	20.4	23.4	26.5	0.85
Thrombectomy	12.2	11.7	10.4	12.2	0.86
Non-IRA PCI	4	5.4	5.7	4.9	0.55
PCI complications (no-reflow, distal embolization, side branch occlusion, artery perforation)	7.3	9.3	14.4	14.6	0.003
Intra-aortic balloon pumping	3.4	4.7	4.3	2.4	0.69

Values are presented as percentages or medians (inter-quartile range)

*IRA* infarct-related artery, *LAD* left anterior descending, *LCX* circumflex artery, *LMCA* left main coronary artery, *PCI* percutaneous coronary intervention, *RCA* right coronary artery

**Fig. 1:**
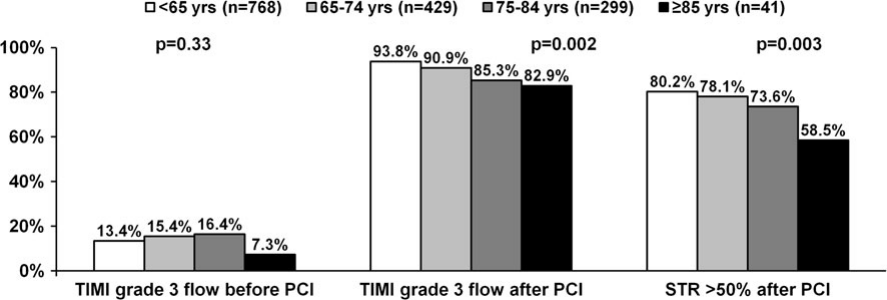
Thrombolysis in myocardial infarction flow before and after PCI, and ST-segment resolution >50 % frequency in 1,537 patients undergoing immediate percutaneous coronary intervention stratified by age. *PCI* percutaneous coronary intervention, *STR* ST-segment resolution, *TIMI* Thrombolysis in myocardial infarction

In the coronary angiography prevalence of multivessel disease increased with age. A total of 1,537 patients (93.2 % of study population) underwent immediate PCI. Elderly patients were more likely to be treated conservatively after coronary angiography and were less likely to receive stents, especially drug-eluting stents during immediate PCI (Table 2). TIMI grade 3 flow frequency before PCI was similar among age groups, but elderly patients were less likely to achieve optimal epicardial flow (TIMI grade 3 flow) after PCI, and were more likely to have PCI complications than their younger counterparts (Fig. 1; Table 2). Similarly, rate of ST-segment resolution >50 % after PCI has shown age-dependency.

**Fig. 2:**
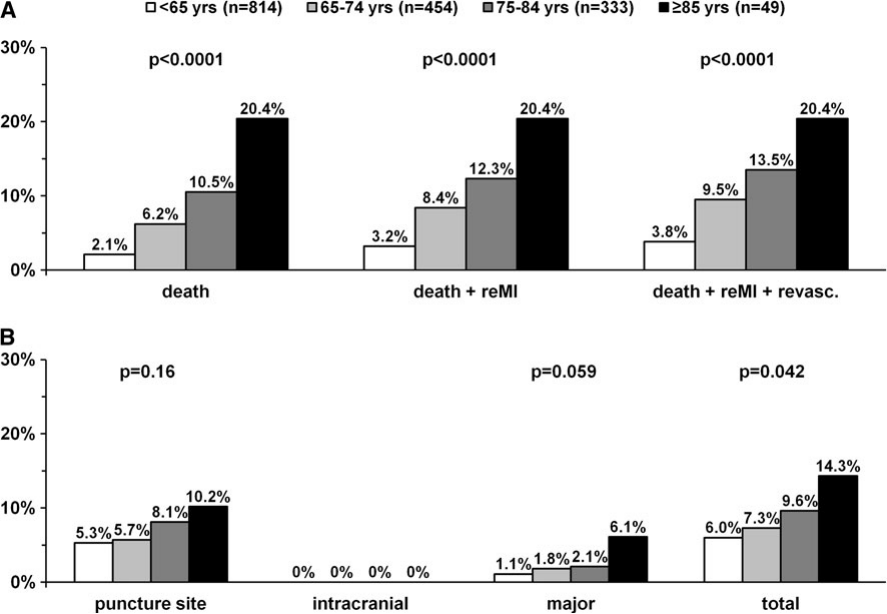
Ischemic events **a** (death, death + reinfarction, death + reinfarction + revascularization) and bleeding events **b** (puncture site hematoma, intracranial hemorrhage, major bleeding requiring transfusion, total bleeding events) at 30-day follow-up stratified by age. *ReMI* reinfarction

**Fig. 3:**
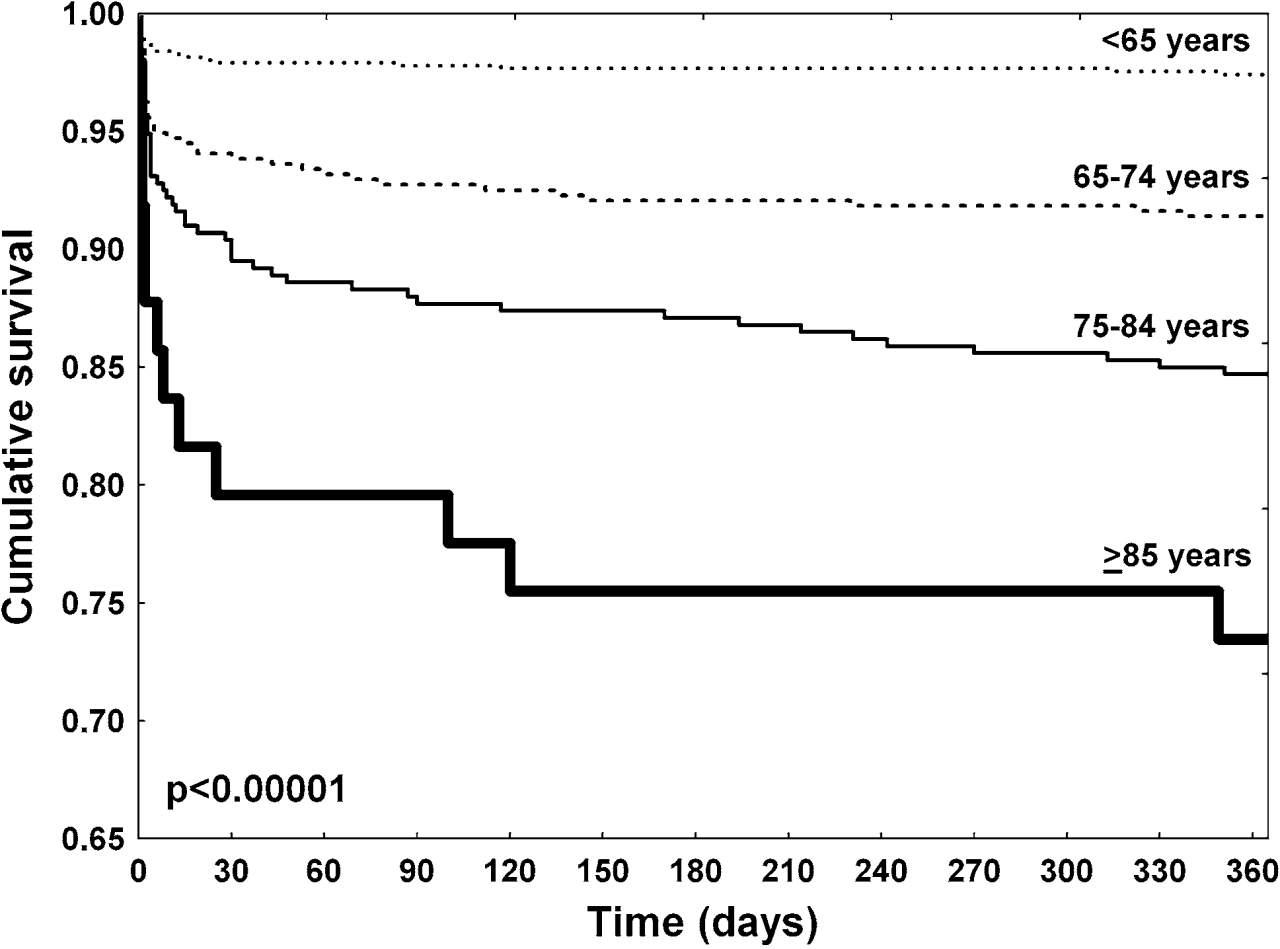
One-year Kaplan–Meier survival curves stratified by age

As shown in Fig. 2a, the rates of death, death + reinfarction and all major adverse cardiovascular events at 30 days were increased with age. In contrast, incidences of reinfarction and urgent revascularization at 30 days were independent of age. The Kaplan–Meier curves for survival according to age are shown in Fig. 3. In Cox regression analysis, independent predictors of 30-day death were: age, diabetes mellitus, previous stroke, heart rate on admission, systolic blood pressure on admission, cardiogenic shock (Killip IV) on admission, diagnosis-to-balloon time, stent implantation during PCI, drug-eluting stent implantation during PCI, non-infarct-related artery PCI, left anterior descending artery as infarct-related artery, TIMI grade 3 flow after PCI, ST-segment resolution >50 % after PCI and major bleeding requiring transfusion (Table 3).

**Table 3:** Multivariate Cox regression analysis for 30-day death

Variable	Hazard ratio	95% Confidence interval	*p* Value
Age (per 1 year)	1.049	1.026–1.072	<0.0001
Diabetes mellitus	1.879	1.074–3.288	0.027
Previous stroke	3.118	1.311–7.416	0.010
Heart rate on admission (per 1 beat/min)	1.025	1.013–1.038	<0.0001
Systolic blood pressure on admission (per 1 mmHg)	0.983	0.973–0.993	0.001
Killip IV on admission	2.681	1.347–5.337	0.005
Diagnosis to balloon time (per 1 min)	0.995	0.990–1.000	0.035
IRA LAD	1.971	1.142–3.399	0.015
Stent implantation during PCI	0.369	0.197–0.689	0.002
Drug-eluting stent implantation during PCI	0.401	0.175–0.918	0.031
Non-IRA PCI	3.055	1.402–6.657	0.005
TIMI grade 3 flow after PCI	0.396	0.221–0.709	0.002
ST-segment resolution >50% after PCI	0.352	0.205–0.602	<0.0001
Major bleeding requiring transfusion	3.057	1.056–8.845	0.039

Values are presented as hazard ratios with 95% confidence intervals

*IRA* infarct-related artery, *LAD* left anterior descending, *PCI* percutaneous coronary intervention, *TIMI* Thrombolysis in myocardial infarction

As clearly shown in Fig. 2b there were no differences in the occurrence of puncture site hematoma and intracranial hemorrhage (which did not occur in either group) across age groups. A trend toward higher risk of major bleeding requiring transfusion and significantly higher incidence of all bleeding complications in elderly patients (especially ≥85 years of age) were observed.

## Discussion

Our study suggests that age is still an important determinant of treatment strategies selection, even in well-organized networks of STEMI treatment. Elderly patients are treated less aggressively in terms of antiplatelet therapy, have experienced longer delays to successful reperfusion, and they are at higher risk of death during follow-up.

In our study, similar to previous studies, and as expected, higher mortality rate was observed in older patients. There are many reasons that contribute to this higher mortality. One of potential explanations of the short- and long-term clinical outcome worsening in elderly STEMI patients is higher prevalence of comorbidities. In line with previous studies, elderly patients were more likely to have diabetes mellitus, previous myocardial infarction, previous heart failure symptoms, previous stroke, and chronic kidney disease [1, 10–14]. Older patients experienced longer time-delays to admission and primary PCI, and presenting more frequently with acute heart failure symptoms. Importantly, ischemia time is a major determinant of survival in patients with STEMI. Another important risk factor for higher mortality in patients with acute coronary syndromes is the presence of renal function impairment [15, 16]. Preexisting impairment of renal function, diabetes mellitus and advanced age are also associated with increased risk of contrast induced nephropathy development after coronary angiography and primary PCI, which may led to worsening of long-term prognosis [17, 18]. Elderly patients are also at higher risk of non-cardiac death during follow-up related to cancer or lung diseases. Similarly, to previous reports in our study observed frequency of reinfarction and need of repeated revascularization was comparable across age groups [1, 10].

It is well established that in elderly patients primary PCI success rate is lower, with higher risk of angiographic complications than in younger counterparts [12–14]. In our study, the frequency of optimal epicardial flow (TIMI grade 3 flow) after primary PCI decreases with age. In contrast, in the Controlled Abciximab and Device Investigation to Lower Late Angioplasty Complications (CADILLAC) Trial correlation between final epicardial flow after PCI and age was not observed [10]. The complex coronary anatomy observed in elderly patients may be associated with a higher incidence of distal embolization, which is a important determinant of myocardial perfusion after primary PCI, as well as long-term clinical outcome [13, 14, 19]. In addition, De Luca et al. have found a relationship between increased age and impaired myocardial perfusion assessed by myocardial blush grade, and ST-segment resolution. Importantly, age and poor myocardial perfusion were independently associated with 1-year mortality [12]. The higher prevalence of multi-vessel disease and the fear of complications among elderly may account for more frequent selection of initial conservative approach, with postponed PCI or coronary artery bypass grafting. Presence of multi-vessel disease in STEMI patients influences the clinical outcomes of patients treated with primary PCI [20]. Also, as confirmed by our study patients with advanced age are less likely to be treated with drug-eluting stents in STEMI setting [21].

In the analyzed patients population there was a trend toward higher rate of major bleeding requiring transfusion in patients with advanced age [10]. Importantly, major bleeding occurrence is a strong predictor of short- and long-term mortality [22–26]. Also, major bleeding may be associated with higher incidence of ischemic events, for example myocardial infarction, unplanned ischemic revascularization, and stent thrombosis [22]. An increased likelihood of the vascular access site complications (hematomas or aneurysm) in elderly patients is a result of the presence of calcified, fragile and bleeding prone vessels in these patients. In addition, in elderly patients frequently reduced kidney function is leading to overdosing of antithrombotic drugs, and to the increased risk of bleeding. Access site bleeding complications could be decreased by broad usage of radial approach, which was used in <15 % of patients in the EUROTRANSFER registry [27]. Safety and efficacy of transradial catheterization in the elderly is similar to observed in younger patients. Importantly, it may improve the comfort of the patients, especially in the context of the age-related diseases that frequently affect elderly patients [28]. However, advanced age was identified as an independent predictor of selection femoral over radial access by the operator during primary PCI in our registry [27]. Also, a fear of bleeding may limit the use of antiplatelet agents, especially glycoprotein IIb–IIIa inhibitors in patients ≥75 years of age [29]. Importantly, previous studies have shown that patients who present with an acute coronary syndrome and do not receive guideline-recommended therapies, including glycoprotein IIb–IIIa inhibitors experienced higher short- and long-term mortality [30–32].

## Limitations of the study

The present study has a number of limitations. First, the study group is relatively small, and the very eldery patient subset (≥85 years of age) comprised only 3 % of the study population. Also, the study focused mainly on 30-day clinical outcomes. Secondly, patients were not screened for contraindications to use of each medication and appropriateness of used dosage was not assessed. It is very likely that in some of patients various therapies were not used due to an important clinical reason. The registry was conducted between November 2005 and January 2007 when new P2Y12 inhibitors (prasugrel, ticagrelor) were not available. Also, the frequency of bivalirudin monotherapy was rather low, as it was recommended as alternative to unfractionated heparin and glycoprotein IIb–IIIa inhibitors combination recently [5]. Thus, the study did not cover most contemporary pharmacological treatment patterns for STEMI. On the other hand, since year 2007 there was no significant change in the recommendations concerning the application of primary PCI in STEMI setting [5]. Finally, the interpretation of the TIMI flow grade measurements, as well as ST-resolution was limited by the fact that these represent not independent core-lab, but physician’s assessments.

## Conclusions

Age was an important determinant of treatment strategies selection and clinical outcomes in the group of consecutive STEMI patients transferred for primary PCI. Further efforts should be made to reduce delays and to optimize treatment of STEMI, regardless of patients’ age.
